# Class A CpG oligodeoxynucleotide inhibits IFN‐γ‐induced signaling and apoptosis in lung cancer

**DOI:** 10.1111/1759-7714.13351

**Published:** 2020-02-17

**Authors:** Shuhei Teranishi, Nobuaki Kobayashi, Seigo Katakura, Chisato Kamimaki, Sousuke Kubo, Yuji Shibata, Masaki Yamamoto, Makoto Kudo, Hongmei Piao, Takeshi Kaneko

**Affiliations:** ^1^ Department of Pulmonology Yokohama City University Graduate School of Medicine Yokohama Japan; ^2^ Respiratory Disease Center Yokohama City University Medical Center Yokohama Japan; ^3^ Department of Respiratory Medicine Affiliated Hospital of Yanbian University Yanji China

**Keywords:** Apoptosis, CpG oligodeoxynucleotide, interferon‐γ, non‐small cell lung cancer, polyguanosine

## Abstract

**Background:**

Currently, anticancer immunotherapy based on PD‐1/PD‐L1 blockade with immune checkpoint inhibitors (ICIs) is being used as a standard therapy for non‐small cell lung cancer (NSCLC). However, more effective treatments are required as these tumors are often resistant and refractory. Here, we aimed to determine the effects of immunomodulatory oligodeoxynucleotides (ODNs) in terms of the presence or absence of CpG motifs and the number of consecutive guanosines.

**Methods:**

Western blots were used to measure the molecules which regulate the expression of PD‐L1 in human lung cancer cell lines after incubation with several cytokines and ODNs. The expression of PD‐L1 and β2‐microglobulin (β2‐MG) on A549 cells, and IFN‐γ‐induced apoptosis with ODNs were examined by flow cytometry. The relationship between IFN‐γ receptor and ODN was analyzed by ELISA and immunofluorescence chemistry.

**Results:**

Our results verified that A‐CpG ODNs suppress the upregulation of IFN‐γ‐induced PD‐L1 and β2‐MG expression. In addition, we found that ODNs with six or more consecutive guanosines (ODNs with poly‐G sequences) may competitively inhibit the IFN‐γ receptor and abolish the effect of IFN‐γ, thereby suppressing apoptosis and indoleamine 2,3‐dioxygenase 1 expression in human lung cancer cells. The tumor microenvironment regulates whether this action will promote or suppress tumor immunity. Thus, in immunotherapy with CpG ODNs, it is essential to consider the effect of ODNs with poly‐G sequences.

**Conclusions:**

This study suggests that ODNs containing six or more consecutive guanosines may inhibit the binding of IFN‐γ to IFN‐γ receptor. However, it does not directly show that ODNs containing six or more consecutive guanosines competitively inhibit the IFN‐γ receptor, and further studies are warranted to confirm this finding.

**Key points:**

- **Significant findings of the study:** Oligodeoxynucleotides with a contiguous sequence of six or more guanosines may competitively inhibit the IFN‐γ receptor and abolish the action of IFN‐γ. This may suppress IFN‐γ‐induced apoptosis and indoleamine‐2,3‐dioxygenase‐1 expression in human lung cancer cells.
- **What this study adds:** A‐CpG and poly‐G ODN may overcome tolerance if the cause of ICI tolerance is high IDO expression. However, IFN‐γ also has the effect of suppressing apoptosis of cancer cells, and it is necessary to identify the cause of resistance.

## Introduction

Lung cancer is the leading cause of cancer‐associated deaths worldwide. Currently, treatment with immune checkpoint inhibitors (ICIs) has improved the prognosis of patients with advanced lung cancer.^1^, ^2^ Only anti‐PD‐1 and anti‐PD‐L1 antibodies as immune checkpoint blockers have significantly improved the prognosis of advanced lung cancer in clinical trials. These observations indicate the importance of the PD‐1/PD‐L1 axis in tumor immunity and highlight the functional suppression of cytotoxic T lymphocytes (CTLs) by the binding of PD‐1 expressed on CTLs to PD‐L1 on tumor cells or antigen‐presenting cells. Thus, anti‐PD‐1 or anti‐PD‐L1 antibodies activate CTLs by inhibiting this binding in the tumor microenvironment.^3^ Compared to existing chemotherapies, anti‐PD‐1 antibody significantly prolonged the overall survival of patients with untreated advanced non‐small cell lung cancer.^4^

PD‐L1 expression is regulated by interferon‐γ (IFN‐γ).^5^ Binding of IFN‐γ to the IFN‐γ receptor activates the JAK/STAT pathway, resulting in enhanced expression of PD‐L1 and β2‐microglobulin (β2‐MG), a component of the major histocompatibility complex (MHC) class I.^6^ These molecules are important for tumor immunity, they are not only the target molecules of ICIs but are also responsible for resistance to anti‐cancer immunotherapy. Some researchers have attempted to overcome this resistance by combining ICIs or combining ICIs with immune stimulants such as TLR3 agonist^7^ or 4‐1BB.^8^ Currently, we are developing immunotherapy using CpG oligodeoxynucleotides (ODNs) for this purpose. Synthetic ODNs have various immunomodulatory effects. Among them, CpG ODNs are ligands of TLR9 expressed on B cells and dendritic cells in humans, and they exert potent immunostimulatory effects via activation of these cells. The immunomodulatory activity differs among the several types of CpG ODNs; A‐CpG ODNs mainly activate plasmacytoid dendritic cell maturation and IFN‐α secretion, while B‐CpG ODNs mainly activate B cells and induce TNF‐α production.^9^ However, little is known regarding the mechanism underlying the effect of CpG ODNs on molecules related to immune checkpoint in human lung cancer.

Here, we investigated the effects of CpG ODNs on the expression of PD‐L1 and β2‐MG in human lung cancer cells. We also determined the effect of CpG ODN on the IFN‐γ/JAK/STAT pathway, which is involved in the expression of PD‐L1 and β2‐MG.

## Methods

### Cell lines and culture

The human lung carcinoma cell lines H226 (squamous cell carcinoma), H460 (large cell carcinoma), H520 (squamous cell carcinoma), and A549 (adenocarcinoma) were purchased from ATCC (Manassas, VA, USA). The cells were maintained in RPMI‐1640 medium (Sigma Aldrich, St. Louis, MO, USA) with 10% FBS (Thermo Fisher Scientific Inc., Waltham, MA, USA) and 1% penicillin‐streptomycin (Gibco, Grand Island, NY, USA). The cells were incubated at 37°C in a humidified atmosphere of 5% CO_2_.

### ODNs and reagents

The sequences of the ODNs used in this study are listed in Table 1. The ODNs were purchased from Sigma Aldrich, Japan (Tokyo, Japan). Poly‐G ODN conjugated to TAMRA for immunofluorescence microscopy was purchased from Sigma Aldrich, Japan. Recombinant human IFN‐γ and IFN‐α were purchased from BioLegend (San Diego, CA, USA). Recombinant human IFN‐β was purchased from Abcam (Cambridge, UK).

**Table 1:** Sequence of synthetic oligodeoxynucleotides (ODNs) used in this study

Name	Sequence (5′ → 3′)
A‐CpG (D35)	GGTGCATCGATGCAGGGGGG
A‐CpG control (D35A)	GGTGCATTGATGCAGAAAAAA
B‐CpG (K3)	GCTAGACGTTAGCGT
B‐CpG control (1612)	GCTAGAGCTTAGCGT
D122	GGTGCATTGATGCAGGGGGG
D122G3	GGTGCATTGATGCAGGG
G6D122	GGGGGGTGCATTGATGCAGG
Poly‐G	GGGGGGGGGGGGGGG
Poly‐A	AAAAAAAAAAAAAAA

### Western blotting

The human lung carcinoma cell lines, H226, H460, H520, and A549 (2 × 10^5^/well) were seeded in six‐well tissue culture plates and incubated for 24 hours. The cells were left untreated or treated with IFN‐γ (10 or 50 ng/mL), IFN‐α (800 U/mL), or IFN‐β (10 ng/mL) and/or ODNs (3 μM), and further cultured for 30 minutes or 16 hours. Then, the cells were lysed in cold cell lysis buffer (Cell Signaling Technology, Danvers, MA, USA) containing protease and phosphatase inhibitors (Cell Signaling Technology) and centrifuged for 10 minutes at 14 000 × *g* at 4°C. Samples containing 30 μg protein were boiled for five minutes, size‐separated on a 10% precast gel (Bio‐Rad, CA, USA), and transferred onto a polyvinylidene difluoride membrane (Thermo Fisher Scientific Inc.). The immunoblots were probed with antibodies specific for JAK1, phosphorylated (p)‐JAK1, JAK2, p‐JAK2, STAT1, p‐STAT1, PD‐L1, β2‐MG, indoleamine 2,3‐dioxygenase 1 (IDO), and β‐actin, followed by probing with anti‐rabbit IgG horseradish peroxidase (HRP)‐linked secondary antibody (Cell Signaling Technology). The signals were visualized with Image Quant LAS 500 (GE Healthcare UK Ltd., Buckinghamshire, England).

### Flow cytometry

A549 cells (2 × 10^5^/well) were seeded in six‐well tissue culture plates and incubated for 24 hours. The cells were left untreated or treated with IFN‐γ (10 ng/mL), IFN‐α (800 U/mL), or IFN‐β (10 ng/mL) and/or ODNs (3 μM) and cultured further for 16 hours. After the cells were collected, centrifuged, and washed, they were incubated for 20 minutes with 5 μL APC‐conjugated PD‐L1 and PECy7‐conjugated β2‐MG and 7‐AAD (BioLegend) and analyzed on a BD FACSCanto II flow cytometer (Becton‐Dickinson, San Jose, CA, USA).

### Immunofluorescence microscopy

#### PD‐L1, β2‐MG, and IDO

A549 cells (8 × 10^4^/well) were seeded in an eight‐well chamber slide (Thermo Fisher Scientific Inc.) and incubated for 24 hours. The cells were left untreated or treated with IFN‐γ (10 or 50 ng/mL) and/or ODNs (3 μM) and cultured further for 16 hours. The cells were fixed with 4% paraformaldehyde for 15 minutes at room temperature and in methanol for 10 minutes at −20°C. Each slide was treated with blocking buffer (3% BSA‐PBS) for one hour at room temperature. Anti‐PD‐L1, anti‐β2‐MG, or anti‐IDO antibody (Cell Signaling Technology) was incubated with cells overnight at 4°C. The cells were incubated with secondary antibody (Alexa 488 anti‐rabbit IgG, Cell Signaling Technology) for one hour at room temperature, and mounted with ProLong Gold antifade reagent with DAPI (Cell Signaling Technology). Each slide was observed under a Keyence BZ‐X800 microscope (Keyence, Osaka, Japan).

#### IFN‐γ receptor and poly‐G ODN‐conjugated TAMRA

A549 cells (8 × 10^4^/well) were seeded in eight‐well chamber slides and incubated for 24 hours. The cells were fixed with 4% paraformaldehyde for 15 minutes at room temperature and in methanol for 10 minutes at −20°C. Each slide was treated with blocking buffer (3% BSA‐PBS) for one hour at room temperature. Anti‐IFN‐γ receptor antibody (Abcam) and poly‐G ODN conjugated‐TAMRA (Sigma Aldrich, Japan) were incubated with cells overnight at 4°C. The cells were incubated for one hour at room temperature with the secondary antibody (Alexa 488 anti‐rabbit IgG, Cell Signaling Technology) for anti‐IFN‐γ receptor antibody detection and mounted with ProLong Gold antifade reagent with DAPI (Cell Signaling Technology). Each slide was observed under the BZ‐X800 microscope (Keyence).

### ELISA

IFN‐γ was detected using the human IFN‐γ ELISA MAX standard set (BioLegend). Plates were coated with a human IFN‐γ capture antibody. Blocking buffer (10% FBS‐PBS) was added to block the remaining protein‐binding sites on the plate. The plates were divided into human IFN‐γ standard only, human IFN‐γ standard, and ODN administration. Human IFN‐γ detection antibody was added, followed by avidin‐HRP, which binds to the biotin‐labeled detection antibody. Tetramethylbenzidine was added, followed by stop solution, and the absorbance was read with an iMark plate reader (Bio‐Rad).

### Apoptosis assay

The effect of ODNs on IFN‐γ‐induced apoptosis was detected using 7AAD and APC‐conjugated annexin V (BioLegend). A549 cells (5 × 10^4^/well) were incubated for 72 hours with IFN‐γ (200 ng/mL) and/or ODNs (3 μM). Subsequently, the cells were collected, centrifuged and washed, incubated for 15 minutes with 5 μL APC‐conjugated annexin V and 7AAD, and analyzed on the BD FACSCanto II (Becton‐Dickinson).

### Statistical analysis

Student's *t*‐tests were used to analyze all the results. *P*‐values <0.05 were considered statistically significant. Statistical analyses were performed using JMP ver. 12 (SAS Institute, Cary, NC).

## Results

### A‐CpG suppresses the expression of PD‐L1 and β2‐MG induced by IFN‐γ in a human lung cancer cell line

**Figure 1:**
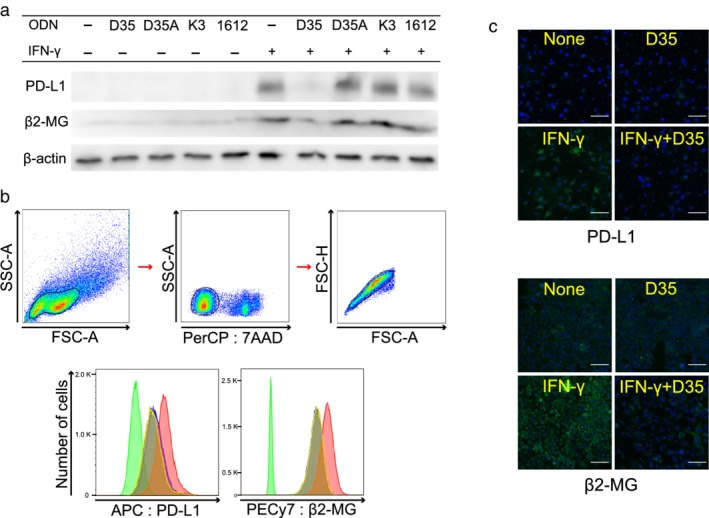
Effect of A‐CpG (D35) or B‐CpG (K3) ODN on the expression of PD‐L1 and β2‐ microglobulin in the A549 cell line. A549 cells were stimulated with or without recombinant human IFN‐γ (10 ng/mL) and/or the indicated ODNs (3 μM), and further cultured for 16 hours. PD‐L1 and β2‐microglobulin expression was detected using western blotting (**a**). D35A was constructed by replacing the consecutive guanosines in the 3′ tail of D35 with consecutive adenosines. ODN 1612, which contains the GpC motif instead of the CpG motif, was the control ODN for B‐CpG. The expression of PD‐L1 or B2‐MG in A549 cells after stimulation with IFN‐γ, A‐CpG (D35), or both was identified using flow cytometry (**b**), and immunofluorescence microscopy. Scale bar = 100 μm (**c**). (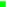) Iso type, (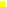) none, (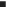) D35, (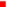) IFN‐y and (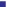) IFN‐y+D35.

A549, a human lung adenocarcinoma cell line, was incubated with A‐CpG ODN (D35), A‐CpG control ODN (D35A), B‐CpG ODN (K3), or B‐CpG control ODN (1612) (3 μM) and/or IFN‐γ (10 ng/mL) for 16 hours. The expression levels of PD‐L1 and β2‐MG were detected using immunoblotting. Although PD‐L1 was not expressed in A549 in the absence of IFN‐γ stimulation after co‐culture with ODNs, the expression of IFN‐γ‐induced PD‐L1 was reduced after co‐culture with A‐CpG (D35) (Fig 1a). Other ODNs did not suppress IFN‐γ‐induced PD‐L1 expression (Fig 1a). The expression of β2‐MG did not change after co‐culture with any ODN; however, the upregulation of β2‐MG induced after IFN‐γ stimulation reduced after coculture with A‐CpG (D35). Other ODNs did not suppress the IFN‐γ‐induced upregulation of β2‐MG (Fig 1a).

Flow cytometry was performed to confirm the effects of A‐CpG (D35) in the A549 lung cancer cell line, and the results showed that the IFN‐γ‐induced expression of PD‐L1 and β2‐MG was reduced after coculture with A‐CpG (D35) (Fig 1b). Fluorescence microscopy also showed that the upregulation of PD‐L1 and β2‐MG after IFN‐γ stimulation was reduced after coculture with A‐CpG (D35) (Fig 1c).

### ODNs with six or more consecutive guanosines suppress the IFN‐γ/JAK/STAT pathway, with or without the CpG motif

**Figure 2:**
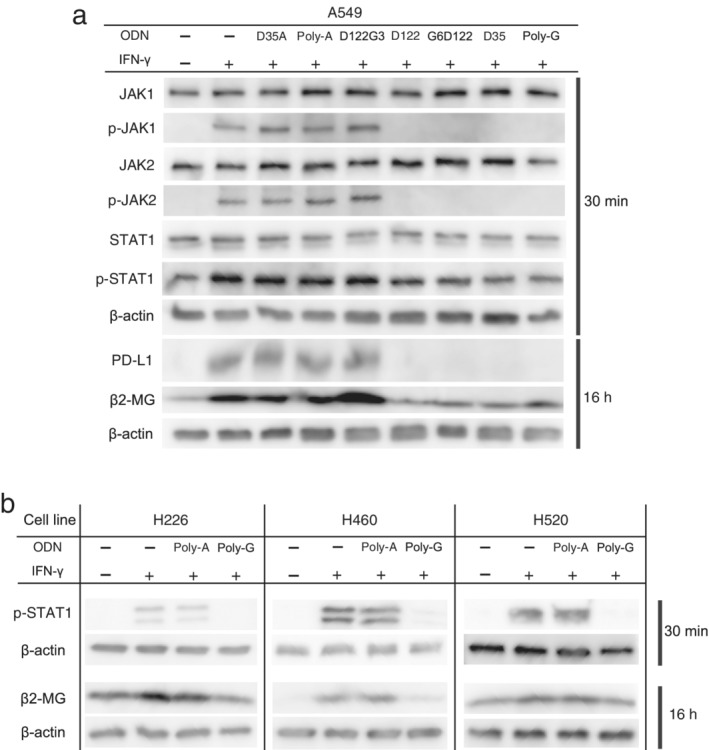
Effect of ODNs with consecutive guanosines on the IFN‐γ/JAK/STAT1 pathway. (**a**) IFN‐γ and the indicated ODNs containing several consecutive guanosines were added to A549 cells. JAK1, phospho‐JAK1, JAK2, phospho‐JAK2, STAT1, and phospho‐STAT1 levels were confirmed after 30 minutes, and PD‐L1 and β2‐microglobulin expression levels were confirmed after 16 hours using western blotting. Poly‐A ODN harbored pure consecutive adenosines. D122, which contains the GpC motif instead of CpG, was the control ODN for A‐CpG (D35). The D122G3 ODN contained three guanosines instead of six guanosines. The G6D122 ODN contained six guanosines in its 5′ head but not in its 3′ tail. (**b**) IFN‐γ and ODNs (poly‐G ODN, poly‐A ODN) were added to H226, H460, and H520 cells. Phospho‐STAT1 levels were confirmed after 30 minutes, and β2‐microglobulin expression levels were confirmed after 16 hours using western blotting.

A‐CpG contains six consecutive guanosines (poly‐G) at the 3′ end in addition to a CpG motif in its sequence. This specific structure in A‐CpG protects it from degradation by DNase and prolongs its half‐life. It was hypothesized that this feature of A‐CpG is responsible for the downregulation of PD‐L1 and β2‐MG, both of which are induced by IFN‐γ stimulation. To identify the sequence responsible for this property of A‐CpG, several ODNs containing different numbers of consecutive guanosines at different locations were generated and their effect on the IFN‐γ/JAK/STAT pathway was assessed using western blotting. In A549 cells, IFN‐γ enhanced the phosphorylation of JAK1, JAK2, and STAT1, and then increased the expression of PD‐L1 and β2‐MG as expected. D122, G6D122, A‐CpG, and poly‐G ODNs abrogated the phosphorylation of molecules downstream in the IFN‐γ signaling pathway and the expression of PD‐L1 and β2‐MG (Fig 2a). In contrast, ODNs without consecutive guanosines (D35A, poly‐A) or those containing only three consecutive guanosines (D122G3) did not show these effects (Fig 2a).

To confirm the effects of poly‐G ODN, human lung cancer cell lines H226 (squamous cell carcinoma), H460 (large cell carcinoma), and H520 (squamous cell carcinoma) were used, and the expression of phosphorylated‐STAT1 and β2‐MG was verified. Similar to A549, phosphorylated‐STAT1 and β2‐MG enhanced by IFN‐γ were suppressed by coculture with poly‐G ODN. Poly‐A ODN did not have this effect (Fig 2b).

### ODNs with consecutive guanosines may competitively inhibit the IFN‐γ receptor

**Figure 3:**
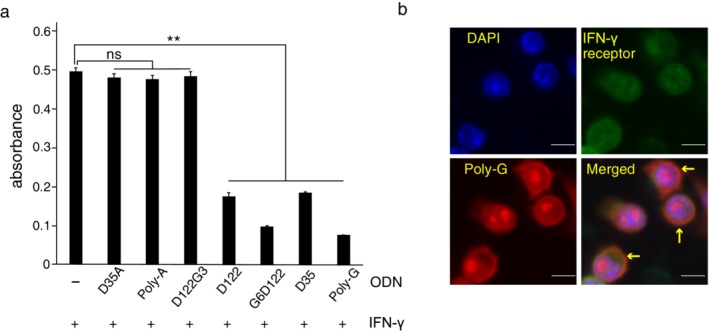
Effect of ODNs with consecutive guanosines on binding of IFN‐γ to its IFN‐γ receptor. (**a**) Recombinant human IFN‐γ and the indicated ODNs were added to a plate coated with IFN‐γ capture antibody; IFN‐γ detection antibody and avidin‐HRP were subsequently added. The binding of IFN‐γ to the IFN‐γ capture antibody was determined from the absorbance value. Results are shown as the means + SD of two experiments. ns, not significant, **P < 0.01 (compared with IFN‐γ administration alone). (**b**) Subcellular localization of IFN‐γ receptor and poly‐G ODN in A549 cells. The localization of the IFN‐γ receptor was detected using GFP fluorescence (green), poly‐G ODN was conjugated with TAMRA (red), and cell nuclei were labeled with DAPI (blue) in A549 cells. Scale bar = 100 μm.

To investigate the inhibitory effect of ODNs harboring the consecutive guanosine motif on the JAK/STAT1 pathway, antibody‐captured IFN‐γ was detected using the sandwich ELISA method. The binding between IFN‐γ and the IFN‐γ capture antibody in the presence of ODNs without guanosine and ODN with three guanosines was not significantly different than that observed in IFN‐γ treatment alone. However, ODNs with six or more guanosines significantly inhibited the binding of IFN‐γ to the IFN‐γ capture antibody (Fig 3a).

Immunofluorescence microscopy was performed to confirm the localization of poly‐G ODN and the anti‐IFN‐γ receptor antibody. When the signals for poly‐G ODN and anti‐IFN‐γ receptor antibody were merged, their localization was consistently observed in the cytoplasm near the nucleus (Fig 3b). These results reveal that ODNs containing six or more consecutive guanosines in their 5′ heads or 3′ tails may inhibit the binding of IFN‐γ to the IFN‐γ receptor and that they reduced the expression of PD‐L1 and β2‐MG by inhibiting the JAK/STAT1 pathway.

### Poly‐G ODN does not affect the JAK/STAT pathway under IFN‐α or IFN‐β stimulation

**Figure 4:**
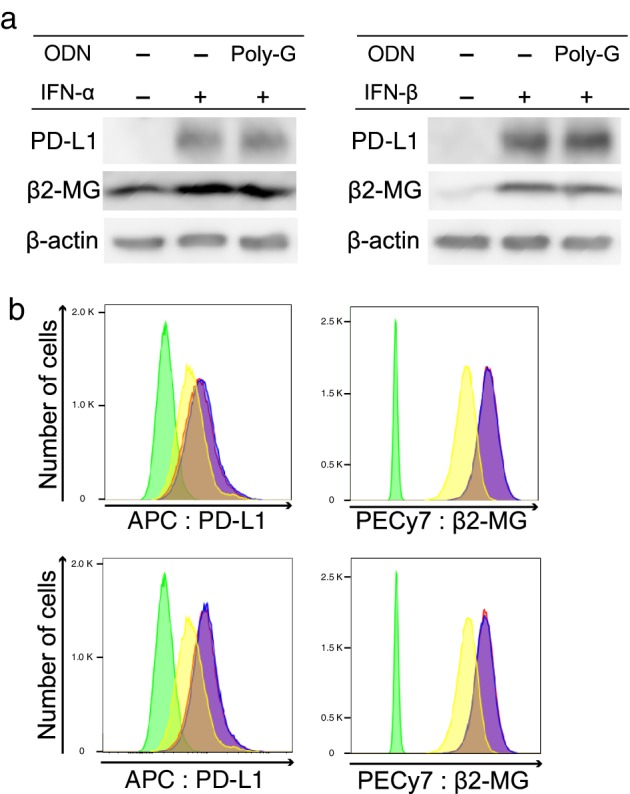
Effect of poly‐G oligodeoxynucleotide (ODN) on the JAK/STAT pathway regulated by IFN‐α or IFN‐β. (**a**) A549 cells were stimulated with recombinant human IFN‐α (800 U/mL) or IFN‐β (10 ng/mL) and/or poly‐G ODN (3 μM), and further cultured for 16 hours. PD‐L1 and β2‐microglobulin expression was assessed using western blotting. (**b**) A549 cells were treated as described above. PD‐L1 and β2‐microglobulin expression was determined using flow cytometry. (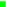) Iso type, (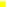) none, (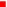) IFN‐α and (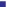) IFN‐α+Poly‐G. (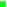) Iso type, (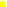) none, (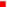) IFN‐β and (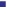) IFN‐β+Poly‐G.

The JAK/STAT1 pathway is a downstream signaling pathway of IFN‐γ as well as type I interferons. Hence, we examined the effect of poly‐G ODN on the JAK/STAT1 pathway activated by IFN‐α or IFN‐β using western blotting and flow cytometry to exclude the possibility of direct inhibition of JAK/STAT1 phosphorylation in cancer cells, and clearly determine the specificity of ODN to IFN‐γ. Similar to that observed with IFN‐γ, the expression of PD‐L1 and β2‐MG was enhanced by IFN‐α (800 U/mL) and IFN‐β (10 ng/mL); however, the expression was not suppressed even after the addition of poly‐G ODN (Fig 4a). Flow cytometry results also showed that poly‐G ODN did not suppress the upregulation of PD‐L1 and β2‐MG by IFN‐α or IFN‐β (Fig 4b).

### ODNs with poly‐G sequences suppress apoptosis and IDO expression induced by IFN‐γ in lung cancer cells

**Figure 5:**
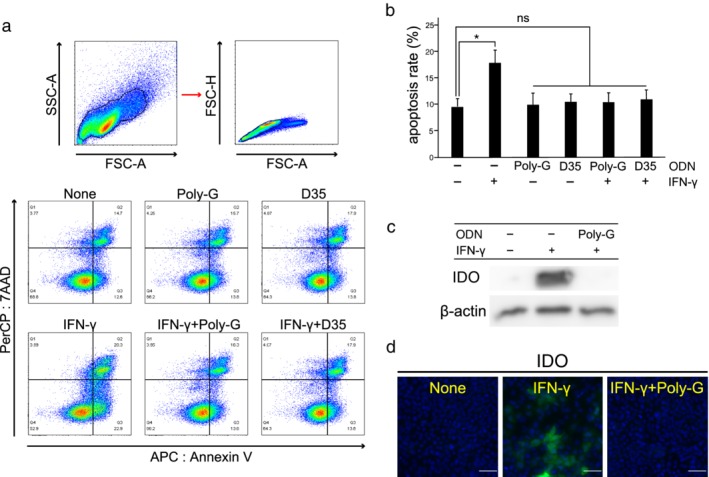
Effect of oligodeoxynucleotides (ODNs) with poly‐G sequences on induction of apoptosis and IDO by IFN‐γ. (**a**,**b**) A549 cells were incubated for 72 hours with IFN‐γ (200 ng/mL) and/or the indicated ODNs with consecutive guanosine sequences (3 μM). Apoptotic cells were detected as annexin V‐positive and 7AAD‐negative cells using flow cytometry. Results are shown as the means + SD of three independent experiments. ns, not significant; * *P* < 0.05 (compared to untreated). (**c**) A549 cells were treated with recombinant human IFN‐γ (50 ng/mL) and/or poly‐G ODN (3 μM), and further cultured for 16 hours. IDO expression was determined using western blotting. (**d**) A549 cells were treated as described above. IDO expression was assessed using immunofluorescence microscopy. Scale bar = 100 μm.

Successful anticancer immune therapy induces the production of inflammatory cytokines in the tumor microenvironment. IFN‐γ plays a central role in suppressing cancer via several mechanisms, including induction of cancer cell apoptosis. A‐CpG (D35) or poly‐G themselves did not affect apoptosis without IFN‐γ (200 ng/mL). Co‐culture of A‐CpG (D35) or poly‐G with IFN‐γ abolished IFN‐γ‐induced apoptosis (Fig 5a,b). Next, we investigated whether an ODN containing consecutive guanosines affected the secretion of immune suppressive enzymes such as IDO and arginase 1 from cancer cells, as the latter is an important mechanism of resistance underlying immune checkpoint blockade. IDO expression was induced after addition of IFN‐γ (50 ng/mL) to A549 cells. However, IFN‐γ action was abrogated and IDO expression was suppressed after addition of poly‐G (Fig 5c,d). These results suggest that A‐CpGs or ODNs containing six guanosines in their 3′ tails suppress the IFN‐γ‐induced pathway as well as IDO expression in lung cancer cell lines.

## Discussion

The effect of CpG ODNs on the expression of PD‐L1 and β2‐MG and on the IFN‐γ/JAK/STAT pathway, which is involved in the expression of PD‐L1 and β2‐MG, in human cancer cell lines was investigated. The results suggest that A‐CpG ODNs suppress the IFN‐γ‐induced expression of PD‐L1 and β2‐MG in human lung cancer by blocking the IFN‐γ receptor and suppressing the phosphorylation of JAK/STAT1, which is regulated by the IFN‐γ signaling pathway. This effect was observed for ODNs containing six or more consecutive guanosines in their tails. These ODNs suppressed the induction of apoptosis of lung cancer cells by IFN‐γ and reduced the expression of IDO, which is a tumor immunosuppressive factor induced in lung cancer cells.

ODNs generally have low cell permeability, and transfection reagents are used for the cellular delivery of nucleic acids. However, previous studies have shown that ODNs with poly‐G sequence can be delivered into tumor cells and human leukocytes without the use of transfection reagents.^10^, ^11^ Therefore, in this study, ODNs containing six or more consecutive guanosines were delivered into the cell membranes simply by incubation with the cultured lung cancer cell lines.

CpG ODNs, which are strong TLR9 agonists, induced tumor regression via the antitumor TLR9‐myeloid differentiation primary response 88 (MyD88) in a murine tumor model.^12^, ^13^, ^14^, ^15^ However, clinical trials have failed to demonstrate the utility of B‐CpG ODNs in patients with malignant tumors.^16^, ^17^, ^18^, ^19^ Hence, we focused on developing novel anticancer immunotherapies using A‐CpG ODNs. Our previous data showed that A‐CpG ODN inhibits tumor growth in a mouse model via poly‐G motif‐mediated T cell activation.^20^ Our present data indicate the possibility of enhancement of antitumor immune responses to A‐CpG ODN via the IFN‐γ pathway in human lung cancer.

IFN‐γ plays a central role in antitumor immunity. IFN‐γ enhances cancer‐specific immune effects on dendritic cells, NK cells, and T cells,^21^ and induces antiproliferation,^22^, ^23^, ^24^ anti‐angiogenesis,^25^, ^26^, ^27^ and proapoptotic effects^28^ against cancer cells. However, it has recently been reported that IFN‐γ can also cause tumor immunosuppression by promoting the expression of PD‐L1, production of IDO, expression of human leukocyte antigen‐G (HLA‐G), a nonclassical MHC class I molecule, and generation of myeloid‐derived suppressor cell (MDSC).^29^ IFN‐γ‐induced IDO degrades and depletes tryptophan, an amino acid necessary for the survival of activated T cells in the tumor microenvironment,^30^ and induces the development of regulatory T cells (Tregs), which also contribute to tumor immunosuppression.^31^, ^32^ The HLA‐G upregulated on tumor cells by IFN‐γ binds to ligands such as immunoglobulin‐like transcript (ILT) 2 and 4 expressed on T cells, suppresses proliferation and cytotoxicity of T cells, and induces Tregs. It also induces immunosuppressive CD4+ and CD8+ T cells when bound to ligands expressed on dendritic cells.^33^, ^34^ IFN‐γ‐induced MDSCs play a major role in the survival and progression of cancer.^35^ In our study, we observed that ODNs containing poly‐G sequences may competitively inhibit the IFN‐γ receptor and abolish the action of IFN‐γ.

This study suggests that ODNs containing six or more consecutive guanosines may inhibit the binding of IFN‐γ to IFN‐γ receptor. However, our study does not directly show that ODNs containing six or more consecutive guanosines competitively inhibit the IFN‐γ receptor, and further studies are warranted to confirm this.

In summary, ODNs containing six or more consecutive guanosines may competitively inhibit the IFN‐γ receptor. Whether this effect supports tumor suppression or growth in the human body depends on the tumor microenvironment in each affected individual. Our observations have provided novel insights into the utilization of CpG ODN‐based immunotherapies, especially those based on poly‐G‐containing A‐CpG ODNs. Further studies are underway for developing A‐CpG ODN‐based therapies against immune checkpoint‐resistant tumors.

## Disclosure

The authors have no conflict of interest.
